# Feasibility of INTACT (INcisionless TArgeted Core Tissue) biopsy procedure for perinatal autopsy

**DOI:** 10.1002/uog.20387

**Published:** 2020-05-01

**Authors:** S. C. Shelmerdine, J. C. Hutchinson, L. Ward, T. Sekar, M. T. Ashworth, S. Levine, N. J. Sebire, O. J. Arthurs

**Affiliations:** ^1^ Department of Clinical Radiology Great Ormond Street Hospital for Children London UK; ^2^ UCL Great Ormond Street Institute of Child Health Great Ormond Street Hospital for Children London UK; ^3^ Department of Histopathology Great Ormond Street Hospital for Children London UK

**Keywords:** autopsy, minimally invasive autopsy, pediatric, perinatal, ultrasound

## Abstract

**Objectives:**

To determine the feasibility and tissue yield of a perinatal incisionless ultrasound‐guided biopsy procedure, the INcisionless Targeted Core Tissue (INTACT) technique, in the context of minimally invasive autopsy.

**Methods:**

Cases of perinatal death in which the parents consented for minimally invasive autopsy underwent postmortem magnetic resonance imaging and an INTACT biopsy procedure, defined as needle biopsy of organs via the umbilical cord, performed under ultrasound guidance. In each case, three cores of tissue were obtained from seven target organs (both lungs, both kidneys, heart, spleen and liver). Biopsy success was predefined as an adequate volume of the intended target organ for pathological analysis, as judged by a pathologist blinded to the case and biopsy procedure.

**Results:**

Thirty fetuses underwent organ sampling. Mean gestational age was 30 weeks (range, 18–40 weeks) and mean delivery‐to‐biopsy interval was 12 days (range, 6–22 days). The overall biopsy success rate was 153/201 (76.1%) samples, with the success rates in individual organs being highest for the heart and lungs (93% and 91%, respectively) and lowest for the spleen (11%). Excluding splenic samples, the biopsy success rate was 150/173 (86.7%). Histological abnormalities were found in 4/201 (2%) samples, all of which occurred in the lungs and kidneys of a fetus with pulmonary hypoplasia and multicystic kidney disease.

**Conclusions:**

Incisionless ultrasound‐guided organ biopsy using the INTACT procedure is feasible, with an overall biopsy success rate of over 75%. This novel technique offers the ideal combination of an imaging‐led autopsy with organ sampling for parents who decline the conventional invasive approach. © 2019 The Authors. *Ultrasound in Obstetrics & Gynecology* published by John Wiley & Sons Ltd on behalf of the International Society of Ultrasound in Obstetrics and Gynecology.

**CONTRIBUTION**

*What are the novel findings of this work?*

Ultrasound‐guided biopsy of organs through the umbilical cord using the INcisionless TArgeted Core Tissue (INTACT) procedure in cases of perinatal death provides a higher biopsy success rate than that reported previously using blinded percutaneous needle biopsy. It is feasible to obtain biopsies from several organs using this technique, avoiding incisions and multiple puncture sites.

*What are the clinical implications of this work?*

This incisionless perinatal biopsy procedure offers the ideal combination of an imaging‐led autopsy with organ sampling for parents who decline a conventional invasive perinatal autopsy approach.

## INTRODUCTION

Non‐invasive perinatal autopsy using postmortem imaging techniques, such as postmortem magnetic resonance imaging (PM‐MRI), demonstrates high concordance rates when compared with conventional invasive autopsy^1^, ^2^, and can offer additional information, especially when neuropathological assessment is non‐diagnostic^3^. Nevertheless, tissue samples are required in some circumstances, such as in macroscopically abnormal organs^4^ or, paradoxically, when all other investigations, including clinical case review and placental assessment, fail to provide an explanation for the cause of death^5^. Tissue sampling permits microbiological, genetic^6^, metabolic^7^ or histological^8^ interrogation, but parents frequently decline conventional autopsy due to the large incisions required to acquire organ material for analysis^9^, ^10^, ^11^.

Minimally invasive autopsy (MIA) methods offer a good compromise, with tissue samples obtained via smaller incisions, usually performed under laparoscopic guidance^12^, ^13^, ^14^, through a single incision, or by means of several needle biopsies through small puncture wounds in the skin^15^. These methods are subjectively more acceptable to parents than is a full autopsy^16^. One study reported a 74.3% consent rate for MIA compared with 77.3% for postmortem imaging alone and 53.8% for conventional autopsy^17^; however, both MIA and conventional autopsy still result in additional incisions to the body. Recent literature has shown perinatal postmortem ultrasound (PMUS) to be a feasible technique for organ visualization^18^, ^19^, although previous studies acquiring tissue following PMUS have demonstrated poor success rates.

In this study, we aimed to improve on previous techniques by assessing the feasibility and yield of a novel technique for obtaining fetal organ tissue samples in which all samples are acquired via the umbilical cord under ultrasound guidance. This method avoids the need for any incisions, thus leaving the patient intact, as the umbilical cord is accessory tissue to the body and would be expected to harden and detach from the body in normal life. We have named this technique the ‘INcisionless TArgeted Core Tissue’ (INTACT) biopsy procedure. If this method proves successful, it could provide parents with an ideal combination of MIA with organ sampling for parents who decline the conventional invasive approach.

## METHODS

Consecutive unselected cases of perinatal death in which the parents consented for MIA by the least invasive approach possible were included in this prospective study over a 2.5‐year period, from 1 July 2017 to 1 January 2019. Exclusion criteria included forensic death or cases without parental consent. This study was approved by a national research ethics committee (REC 09/H0713/2) and all samples were handled in accordance with the Human Tissue Act (2004). Written consent was obtained in each case.

### Preprocedural imaging

Preautopsy 1.5‐T PM‐MRI was performed in all cases according to local departmental protocols^20^. The PM‐MRI results were reported by specialist pediatric radiologists with expertise in postmortem imaging (O.J.A., 10 years' experience; S.C.S., 3 years' experience) and discussed with the pathologist responsible for the case prior to autopsy. Routine external examination of the body was performed. Placental examination was performed as part of the fetal autopsy, when available. The brain was extracted only when clinically indicated (based on either antenatal history or abnormality on PM‐MRI^21^), and with appropriate parental consent. Tissue biopsies were obtained after discussion with the pathologist in charge of the case.

### INTACT biopsy procedure

Postmortem ultrasound was performed by a pediatric radiology research fellow (S.C.S.), according to published guidelines^19^, to assess whether the target organs for biopsy could be visualized. The umbilical clamp was removed if possible, and the umbilical cord cut short, to a length of < 2 cm. A 13.5‐G coaxial needle was inserted through the umbilical cord and, under ultrasound guidance, directed towards the intended target organ. The trocar was removed and a 14‐G Temno Evolution biopsy needle (CareFusion, San Diego, CA, USA) was inserted through the coaxial needle. In order to replicate the standard autopsy procedure, tissue samples were taken from the heart, both lungs, both kidneys, liver and spleen, as per the guidelines of the Royal College of Pathologists^22^, ^23^.

**Figure 1:**
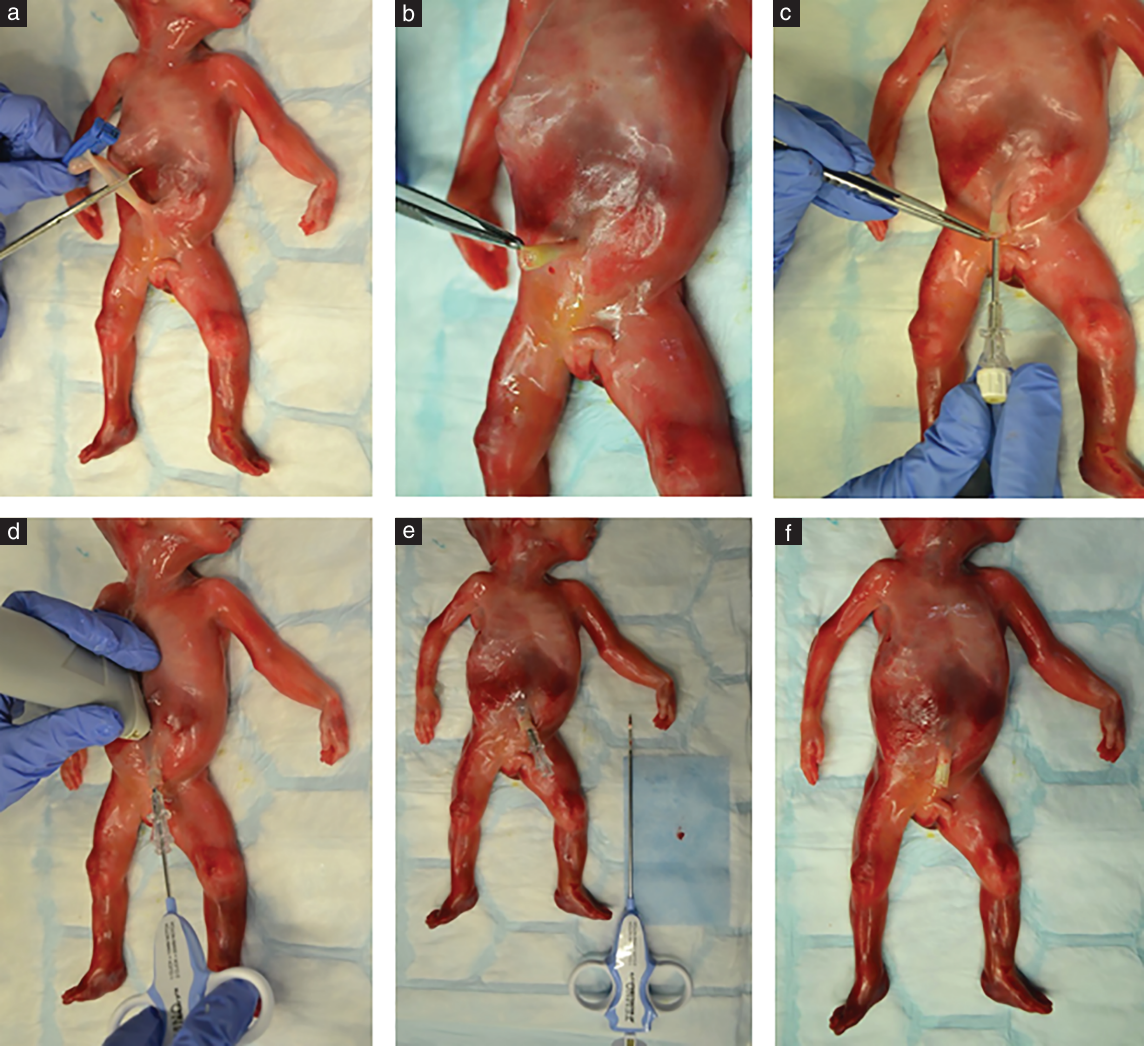
Images demonstrating steps of INcisionless TArgeted Core Tissue (INTACT) biopsy procedure, performed in 20‐week fetus. (a) After review of autopsy consent form, case identification and ultrasound examination, umbilical clamp is removed if possible, and umbilical cord cut to length of < 2 cm. (b,c) 13.5‐G coaxial needle and trocar are inserted via umbilical cord. (d) Once needle is identified on ultrasound to be intra‐abdominal, ultrasound is used to guide it towards intended target organ; trocar is then removed and 14‐G Temno biopsy needle is inserted through coaxial needle. (e) Three cores are obtained per target organ, and tissue samples are placed on blue blotting paper. (f) After procedure, there are no incisions or visible damage to body.

Three core biopsies were acquired per organ, and the coaxial needle and trocar were repositioned for each organ. The same cutting biopsy needle was reinserted each time to obtain all tissue samples. Figure 1 demonstrates the technique step by step and shows the pre‐ and postprocedural cosmetic outcomes.

The entire procedure took approximately 20 min to complete. The performing radiologist was assisted by a mortuary assistant during each procedure, who labelled and collated the tissue samples. The samples were all fixed immediately in formalin, embedded within paraffin blocks and sectioned by histopathology laboratory staff for subsequent evaluation by in‐house pediatric pathologists. No pathologists were present during the INTACT procedures. The sampling order of organs was pseudo‐randomized for each case.

If sampling of the intended organ was unsuccessful due to either tissue autolysis or failure to survive histological processing, but the preceding PM‐MRI demonstrated normal findings, no further action was taken given the low chance of pathological yield^4^. If the sampling of the intended organ was not attempted or tissue biopsy was insufficient, the pathologist determined whether or not to perform further sampling via a laparoscopic approach^14^.

### Histological assessment

One of two perinatal pathologists (J.C.H., N.J.S.), blinded to antenatal history and the sampled target organ, assessed the histology slides in all cases. The slides were reviewed approximately 6 months after the initial biopsy procedure had taken place to eliminate any possible recall bias from clinical reporting of the samples or possible departmental discussion of the autopsies, during either multidisciplinary meetings or professional discussions.

The pathologist was asked to assess each sample for the following criteria: (1) the type of organ tissue in the sample (e.g. heart, liver, etc.), or whether the degree of autolysis precluded such assessment; (2) adequacy of tissue volume (i.e. would the amount of tissue provided be suitable for making a diagnosis, using a subjective score of either ‘yes’ or ‘no’?); (3) presence of pathology (i.e. normal tissue *vs* pathological finding).

### Data analysis

The primary outcome was the rate of successful biopsy using the INTACT procedure. Successful biopsy was predefined as obtaining adequate material of the intended target organ for the reporting pathologist to make a diagnosis (either normal or abnormal). Biopsy failure was predefined as insufficient material for comment, tissue sample too small to undergo histological processing or target organ not sampled. Autopsy results and histological findings were analyzed according to successful organ biopsy and specific organ pathologies, using Microsoft Excel (Microsoft Corp., Redmond, WA, USA).

## RESULTS

### Study cohort

In total, 30 fetuses underwent an INTACT biopsy procedure. Case demographics are outlined in Table 1. Mode of death included nine (30%) terminations of pregnancy, five (16.7%) miscarriages and 16 (53.3%) intrauterine deaths. Fifteen (50%) fetuses were described by the attending pathologist as being markedly macerated, three (10%) as moderately macerated, eight (26.7%) as mildly macerated and four (13.3%) as not macerated. The cause of death on the final autopsy report was fetus‐related anomalies in seven (23.3%) cases, placenta related in seven (23.3%; three due to placental vasculopathy, two due to antepartum hemorrhage and two relating to a cord accident and knot entanglement), ascending maternal genital tract infection in three (10%) and undetermined in 13 (43.3%). Of the seven cases of fetus‐related anomalies, all were suspected as being abnormal on prenatal ultrasound and subsequently underwent termination of pregnancy. The anomalies included: one case of antenatally diagnosed trisomy 18 with large ventricular septal defect; one case of bilateral multicystic dysplastic kidneys with pulmonary hypoplasia; one case of fetal hydrops with talipes (cause unknown); one case of antenatally diagnosed pulmonary atresia with bilateral renal agenesis; two cases of complex fetal brain abnormalities (callosal and neuronal migration anomalies); and one case of hypoplastic feet, thought to be part of amniotic band syndrome.

**Table 1:** Characteristics in 30 cases of perinatal death undergoing INcisionless TArgeted Core Tissue (INTACT) biopsy procedure

Characteristic	Value
Gestational age (weeks)	30; 33 (18–40)
Male fetal sex	17 (56.7)
Postmortem weight (g)	1582; 1380 (98–4060)
Crown–heel length (cm)	38; 41 (20–52)
Crown–rump length (cm)	27; 30 (15–38)
Delivery‐to‐PM‐MRI interval (days)	8; 7 (4–21)
Delivery‐to‐INTACT biopsy interval (days)	12; 11 (6–22)

Data are given as *n* (%) or mean; median (range).

PM‐MRI, postmortem magnetic resonance imaging.

### Tissue biopsy: non‐sampling and additional samples

During the study, there were six cases in which the intended organ for biopsy was not sampled. The reasons for this included: two cases of larger fetuses in which both lungs could not be reached by the biopsy needle, as the distance between the lungs and umbilicus was greater than the length of biopsy needle (11 cm); one case in which the heart could not be reached by the biopsy needle; two cases in which the spleen was not visible on ultrasound for targeting; and one case with bilateral renal agenesis. In the cases in which the lungs and heart could not be sampled by needle biopsy, a decision was made by the pathologist to proceed with laparoscopically assisted MIA to acquire the tissue samples. None of these subsequent samples yielded abnormal tissue.

In one case, additional biopsies were obtained from a neck mass in a 25‐week fetus with known complex fetal brain malformation. These biopsies were conducted after discussion with the pathologist in charge of the case, and with parental consent. They were performed under ultrasound guidance, via a small 1‐cm incision in the skin, not using the INTACT biopsy procedure. The mass demonstrated abnormal mature tissue elements, and a differential diagnosis of a possible hamartoma or teratoma was suggested.

### Biopsy tissue type and yield of sampling

**Figure 2:**
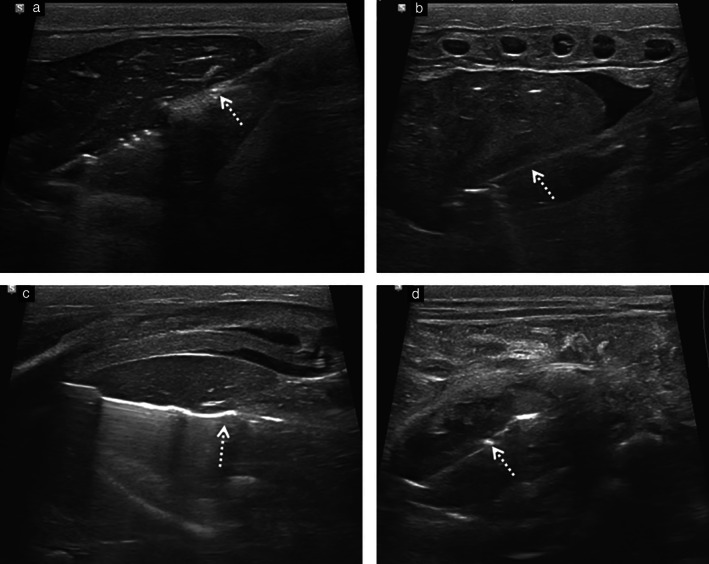
Postmortem ultrasound images in 35‐week fetus during INcisionless TArgeted Core Tissue (INTACT) biopsy procedure. (a) Longitudinal view of liver. (b) Longitudinal view of heart. (c) Longitudinal view of spleen. (d) Transverse view of right kidney. Biopsy needle (dotted arrow) is seen clearly on all images.

The INTACT biopsy procedure took between 20 and 30 min in each case. In total, 201 organs were sampled. There was satisfactory sonographic biopsy needle visualization during all organ sampling (Figure 2). A summary of biopsy success rates, with regards to obtaining both the correct target organ and adequate sample volume, is provided in Table 2. Successful biopsy was achieved in 76.1% (153/201; 95% CI, 69.8–81.5%) of all samples. The highest biopsy success rates were observed for the left lung (28/28, 100%; 95% CI, 85.7–100%) and heart (27/29, 93.1%; 95% CI, 76.9–99.2%), with the spleen having the lowest success rate (3/28, 10.7%; 95% CI, 2.9–28.0%). When excluding the splenic samples from the overall results, the biopsy success rate was 86.7% (150/173; 95% CI, 80.8–91.0%).

**Table 2:** Histological sampling success and tissue yield in 30 fetuses undergoing INcisionless TArgeted Core Tissue (INTACT) biopsy procedure

Organ	Samples (*N*)	Target organ		Tissue type in negative sample	Sample volume		Successful biopsy (*n*/*N* (%) (95% CI))	Pathological diagnosis
		Positive (*n*/*N* (%) (95% CI))	Negative (*n*/*N* (%) (95% CI))		Sufficient (*n*/*N* (%) (95% CI))	Insufficient (*n*/*N* (%) (95% CI))		
Right lung	28	27/28 (96.4) (81.0–99.9)	1/28 (3.6) (0.01–19.2)	1 NDT	23/27 (85.2) (66.9–94.7)	4/27 (14.8) (5.3–33.1)	23/28 (82.1) (63.9–92.6)	1 pulmonary immaturity
Left lung	28	28/28 (100) (85.7–100)	0/28 (0) (0–14.3)		28/28 (100) (85.7–100)	0 (0) (0–14.3)	28/28 (100) (85.7–100)	1 pulmonary immaturity
Heart	29	27/29 (93.1) (81.0–99.9)	2/29 (6.9) (0.9–23.0)	2 liver	27/27 (100) (85.2–100)	0 (0) (0–14.8)	27/29 (93.1) (76.9–99.2)	—
Liver	30	26/30 (86.7) (69.7–95.3)	4/30 (13.3) (4.7–30.3)	4 NDT	25/26 (96.2) (79.6–99.9)	1/26 (3.9) (0–20.5)	25/30 (83.3) (65.6–93.1)	—
Right kidney	29	26/29 (89.7) (72.8–97.2)	3/29 (10.3) (2.8–27.2)	1 NDT, 1 liver tissue, 1 myocardial tissue	22/26 (84.6) (65.9–94.5)	4/26 (15.4) (5.5–34.2)	22/29 (75.9) (57.6–88.1)	1 multicystic kidneys
Left kidney	29	26/29 (89.7) (72.8–97.2)	3/29 (10.3) (2.8–27.2)	1 NDT, 2 fibrous tissue and bowel	25/26 (96.2) (79.6–99.9)	1/26 (3.9) (0–20.5)	25/29 (86.2) (68.8–95.1)	1 multicystic kidneys
Spleen	28	5/28 (17.9) (7.4–36.1)	23/28 (82.1) (63.9–92.6)	6 NDT, 7 muscle, 3 bowel, 3 myocardial tissue, 2 lung, 1 liver, 1 fibrous tissue	3/5 (60.0) (22.9–88.4)	2/5 (40.0) (11.6–77.1)	3/28 (10.7) (2.9–28.0)	—
Overall	201	165/201 (82.1) (76.2–86.8)	36/201 (17.9) (13.2–23.8)	—	153/165 (92.7) (87.6–95.9)	12/153 (7.8) (4.4–13.3)	153/201 (76.1) (69.8–81.5)	—
Overall, excl. spleen	173	160/173 (92.5) (87.5–95.7)	13/173 (7.5) (4.3–12.5)	—	150/160 (93.8) (88.8–96.7)	10/160 (6.3) (3.3–11.3)	150/173 (86.7) (80.8–91.0)	—

excl., excluding; NDT, non‐diagnostic tissue.

The sampling failure rate was 23.9% (48/201), which included 36 cases in which the intended target organ was not present in the biopsy sample and 12 in which there was an insufficient volume of tissue for pathological assessment. The spleen was the target organ that was most frequently not present in the final biopsy sample (23/36, 63.9%). In approximately one‐third (13/36, 36.1%) of cases that did not contain the intended target organ tissue, the pathologist was unable to identify the tissue origin (reported ‘non‐diagnostic tissue’).

### Pathological diagnoses

**Figure 3:**
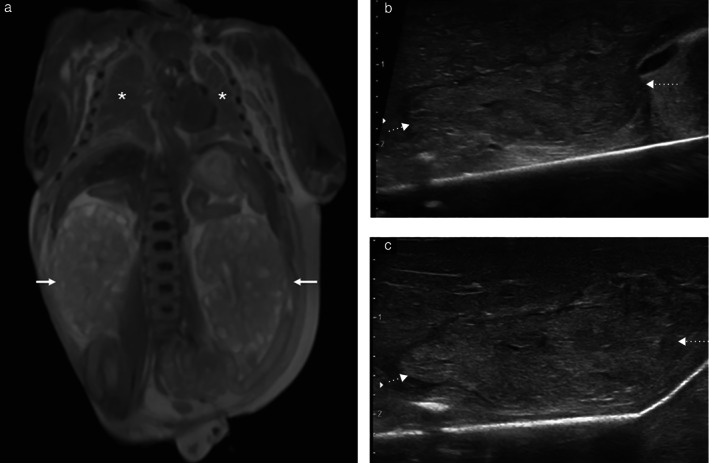
Postmortem magnetic resonance imaging and ultrasound images in 20‐week fetus. (a) Coronal T2‐weighted image of thorax and abdomen demonstrating enlarged cystic kidneys (arrows) with small hypoplastic lungs (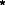). (b,c) Longitudinal postmortem ultrasound views of right and left kidney, respectively. Kidneys are enlarged and lack normal corticomedullary differentiation, making them hard to visualize. Dotted arrows denote upper and lower poles of each kidney.

**Figure 4:**
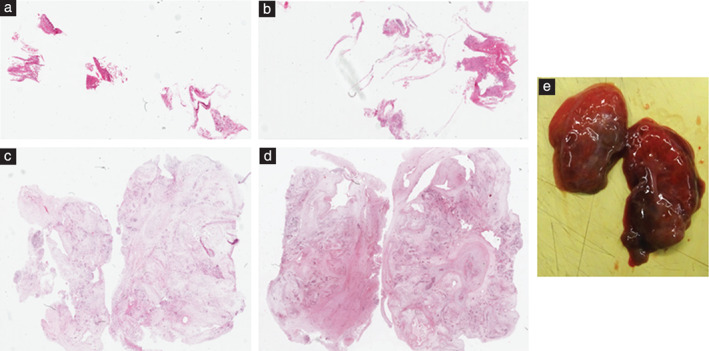
(a–d) Histological samples (with hematoxylin and eosin staining) of right (a,c) and left (b,d) kidneys in 20‐week fetus with multicystic dysplastic kidneys, obtained by INcisionless TArgeted Core Tissue (INTACT) biopsy procedure (a,b) and laparoscopically assisted minimally invasive autopsy (c,d). Samples obtained by laparoscopically assisted procedure were much larger in volume and allowed cystic spaces and scant gloms to be seen. (e) Right and left kidneys in same fetus following removal via 2‐cm subcostal incision during laparoscopically assisted procedure.

The majority of the 153 successful biopsies were of normal tissue, with 17/153 (11.1%) showing some maceration‐related changes. A pathological finding was present in 4/153 (2.6%) biopsy samples, all of which originated from the same 20‐week fetus. This pregnancy had been terminated due to prenatal identification of bilateral enlarged cystic kidneys and pulmonary hypoplasia. There were clear abnormalities on PM‐MRI and ultrasound examination (Figure 3). Both lung biopsy samples demonstrated premature pulmonary development on histology. Both renal biopsy samples contained multiple areas of cystic change but, as they did not include any glomeruli, it was not possible on blinded pathological examination to be certain that these originated from the kidneys; these samples were therefore classified as inadequate. Laparoscopically assisted minimally invasive autopsy was subsequently performed, via a 2‐cm subcostal incision, and the kidneys were removed *en bloc*. The larger volume of tissue obtained from the latter procedure allowed for better pathological assessment (Figure 4).

## DISCUSSION

This study demonstrates the feasibility of an incisionless perinatal needle biopsy procedure, the INTACT biopsy procedure, utilizing ultrasound guidance via a single umbilical entry route. When excluding splenic samples from the overall results, the biopsy success rate was > 85%. This novel incisionless technique has the potential to offer parents an alternative procedure when they wish to avoid invasive techniques but require organ sampling for histopathological, molecular, genetic or metabolic analysis.

Our study has several clinical implications, primarily reduced cost and better accessibility compared with some alternative published MIA techniques. Research groups working in adult postmortem imaging have shown promising early results using CT‐guided biopsies^24^, ^25^, ^26^; however, these are less accessible and applicable to fetuses given their inherent poor tissue contrast and small size^2^. Ultrasound‐guided interventional techniques could be adopted by fetal medicine doctors or obstetricians, as some are already familiar with ultrasound imaging and needle biopsy methods^27^, ^28^, ^29^. This would ideally be combined with rapid feedback from pathologists regarding tissue sample adequacy. Providing parents with a MIA option that avoids any incisions could increase the uptake rate and acceptability^16^, ^30^, ^31^, providing more information for future pregnancy counseling over imaging alone, and feedback for medical audit, training and teaching^32^. Finally, the portability and affordability of the equipment (both ultrasound machine and biopsy needles) make it ideal for use in the developing world, in which microbiological assessment is key to understanding the burden of infectious diseases^31^, ^33^, ^34^, ^35^, ^36^. When implementing this technique, it would also need to be paired with facilities for tissue analysis so that the results can be interpreted in a timely fashion.

Compared with similar studies using needle biopsy methods (Table S1), the INTACT technique has one of the highest biopsy success rates for sampling the heart, lung and renal tissues, but a low yield for splenic tissue (11%). These success rates are likely to be due to a larger‐bore needle and ultrasound visualization of the target organ. Nevertheless, even without these benefits, some studies have reported greater splenic tissue sampling rates (between 0% and 31%^27^, ^29^, ^37^, ^38^, ^39^) than in the present study, although the absolute success rate was lower than for other organs. This may relate to the smaller fetal spleen size during development^40^, with the left lobe of the liver occupying much of the left upper quadrant, and the similarity in sonographic echogenicity of splenic tissue to liver, making differentiation difficult. Other studies in which splenic sampling rates were considerably higher were performed in adults^35^ (in whom the spleen is larger) or extracted organs *en bloc* via an abdominal incision (e.g. laparoscopic technique)^14^. In our view, low splenic tissue yield may not be clinically relevant as splenic tissue rarely contributes towards the cause of pregnancy loss^4^ and, in rare situations in which splenic tissue is important (i.e. in metabolic, infectious and hematological diseases), there is typically splenomegaly, potentially making sampling easier^41^, ^42^.

Although we targeted seven key organs for sampling, we did not include all of the organs in the Royal College of Pathologists' autopsy guidelines^22^, ^23^, ^43^, which include the thymus, thyroid, adrenal glands, intestines and pancreas. The reasons for this are 2‐fold: firstly, sampling the thymus and thyroid would require an incision to the neck, which we wished to avoid, and, secondly, recent evidence from a cohort of 1064 perinatal deaths did not find any cases in which examination of the aforementioned organs provided the cause of death at autopsy^4^. The brain was also not examined or biopsied as part of the INTACT procedure, since further assessment usually requires whole‐brain extraction by a specialist perinatal neuropathologist for expert sectioning and processing, and tissue biopsy samples are typically non‐contributory.

This study has several limitations. The small sample size and lack of full autopsy correlation meant we could not calculate a robust diagnostic accuracy rate, which will be required for future studies, particularly if we are to address fully our false‐negative rates and provide a strong evidence basis with which to consent parents and define the contribution of the technique in providing a cause of death. Nevertheless, in this study, our intention was to first demonstrate feasibility of this novel biopsy technique. As the majority of our cases yielded adequate, normal tissue, we were unable to assess the adequacy of sampling for abnormal pathology in each organ. We also found that our standard biopsy needle length was too short in two larger fetuses to target the lungs and heart using the umbilical approach; thus, a range of longer needles may be required in future. Maceration affected the quality of the samples in our study, with moderate or marked maceration in two‐thirds of cases, which may have resulted in a higher inadequate sampling rate. Biopsy success rates may therefore be improved in the future by preferentially recommending non‐macerated cases for INTACT sampling or counseling parents regarding the possible lower yield in macerated fetuses^44^.

Whilst we did not select our cases based on any predefined criteria other than parental consent for a minimally invasive approach, our cohort did contain a large number of stillbirths and intrauterine deaths. This may have introduced some bias, such as a potentially lower likelihood of abnormal fetal pathologies, but the final causes of death were similar to those in large unselected fetal autopsy series (approximately 50% undetermined, 20% related to fetal anomalies and 30% related to placental and maternal pathologies)^45^, ^46^. In addition, there was wide variation in time from delivery to autopsy. This was not specific to the undertaking of the INTACT procedure, as it is also variable for conventional autopsies in our center given that we receive referrals from different maternity units in our region and do not have a maternity unit on site. Nevertheless, there is good‐quality published evidence that this time interval has no effect on postmortem bacteriological yield^47^ or DNA quality^48^. With further work in a larger cohort of cases with autopsy correlation and a more streamlined protocol pathway, we hope that this interval can be reduced and we can determine better the diagnostic accuracy and best indications for this novel technique. With regards to the immediate future, this technique is feasible and would be possible to carry out for tissue sampling in cases in which parents decline conventional autopsy.

In conclusion, our novel incisionless biopsy procedure (INTACT procedure) is feasible and allows adequate sampling for the majority of organs. This technique offers a potential alternative approach for organ sampling when parents decline the conventional invasive approach.

## Supporting information


**Table S1** Histological sampling success rates reported in adult and pediatric studies using minimally invasive tissue sampling methods
