# Supplementary material for: Feasibility of INTACT (INcisionless TArgeted Core Tissue) biopsy procedure for perinatal autopsy
Source: Ultrasound Obstet Gynecol. 2020 May 1;55(5):667–75. doi: 10.1002/uog.20387 (PMC7317589; doi:10.1002/uog.20387)
Supplement: Supplementary file 1 — Table S1 Histological sampling success rates reported in adult and pediatric studies using minimally invasive tissue sampling methods [file UOG-55-667-s001.docx]

**Table S1** Histological sampling success rates reported in adult and pediatric studies using minimally invasive tissue sampling methods

| **Publication** | **Country** | **n** | **Population** | **Age Range** | **Biopsy Type** | **Needle Gauge** | **Incisions, technique, operator** | **Biopsy Success Rate (%)** | | | | | |
| --- | --- | --- | --- | --- | --- | --- | --- | --- | --- | --- | --- | --- | --- |
|  |  |  |  |  |  |  |  | **Heart** | **Lungs** | **Liver** | **Kidney** | **Spleen** | **Overall** |
| Breeze ACG, 2008 | Cambridge,  U.K. | 29 | Perinatal | 16 – 39 wks GA | Blind, percutaneous | 16-18G Temno cutting needle | Multiple needle puncture sites,  Use of surface landmarks  Performed by obstetrician | 52 | 86 | 76 | 34 | 17 | 53 |
| Garg S, 2009 | Chandigarh, India | 25 | Perinatal | 33 wks GA - 28 days | Blind, percutaneous | Tru-cut needle (gauge not specified) | Multiple needle puncture sites,  Use of surface landmarks  Performed by pathologist | N/A | 84 | 92 | 40  56 – L  24 - R | 20 | 57 |
| Bansal MG,  2012 | Chandigarh, India | 50 | **Adult** | **ADULT**  12 – 80 years | Blind, percutaneous | Needle biopsy gauge not specified | Multiple needle puncture sites,  Use of surface landmarks  Performed by pathologist | 28 | 90 | 82 | 48 | 22 | 54 |
| Celiloglu OS, 2013 | Adana,  Turkey | 76 | Perinatal | 35 wks GA (mean), range not given | Blind, percutaneous | 16G Tru-cut | Multiple needle puncture sites,  Use of surface landmarks  Performed by paediatrician | 43 | 50 | 78 | 0 | 0 | 34 |
| Castillo P, 2015 | Maputo, Mozambique | 30 | **Adult** | **ADULT**  17 – 76 years | US initially to locate organs, then percutaneous needle biopsy | 14G Monopty | Multiple needle puncture sites,  Use of surface landmarks  Performed by pathologist | 80 | 100 | 100 | 67 | 70 | 86 |
| Bassat Q, 2017 | Maputo, Mozambique | 54 | Childhood | 1 month – 15 years | US first to locate organs, then percutaneous needle biopsy | 14G Monopty | Multiple needle puncture sites,  Use of surface landmarks  Performed by pathologist | 47 | 98 | 100 | 56 | 31 | 48 |
| Hutchinson JC, 2019 | London,  U.K. | 91 | Perinatal & Childhood | 15 wks GA – 14 years | Laparoscopic assisted | N/A – scalpel, forceps and scissors as needed | Single epigastric incision, 1-2cm size  Direct visualisation of organs  Performed by pathologist | 100 | 100 | 97 | 100 | 93 | 98 |
| **Current study** | **London,**  **U.K.** | **30** | **Perinatal** | **18 – 40 wks GA** | **Ultrasound guided** | **14G Temno cutting needle** | **No incisions, needle via umbilicus**  **Organ visualised with US guidance**  **Performed by radiologist** | **93** | **92** | **83** | **81** | **11** | **76** |

L, left; R, right; US, ultrasound.
